# 1388. Patients´ perceptions on barriers that prevent and promote the use of telemedicine for HIV care in the public health system of Buenos Aires

**DOI:** 10.1093/ofid/ofad500.1225

**Published:** 2023-11-27

**Authors:** Tomas Kierszenowicz, Manuela Bullo, Maria C Acosta, Maria Jose Rolon, Graciela Oshiro, Diego Cecchini, Claudia Gabriela Rodriguez, Pablo Scapellato, Edgardo Gabriel Bottaro, Marcelo H Losso

**Affiliations:** Consejo Nacional de Investigaciones Científicas y Técnicas, Buenos Aires, Ciudad Autonoma de Buenos Aires, Argentina; Hospital J.M. Ramos Mejía, Buenos Aires, Ciudad Autonoma de Buenos Aires, Argentina; Hospital J.M. Ramos Mejía, Buenos Aires, Ciudad Autonoma de Buenos Aires, Argentina; Hospital Dr. Juan A. Fernández, Buenos Aires, Ciudad Autonoma de Buenos Aires, Argentina; Hospital Dr. Juan A. Fernández, Buenos Aires, Ciudad Autonoma de Buenos Aires, Argentina; Hospital Dr. Cosme Argerich, Buenos Aires, Ciudad Autonoma de Buenos Aires, Argentina; Hospital Dr. Cosme Argerich, Buenos Aires, Ciudad Autonoma de Buenos Aires, Argentina; Hospital Donación Francisco Santojanni, Ciudad Autonoma de Buenos Aires, Ciudad Autonoma de Buenos Aires, Argentina; Hospital Donación F. Santojanni., Buenos Aires, Ciudad Autonoma de Buenos Aires, Argentina; Hospital JM Ramos Mejía, Buenos Aires, Ciudad Autonoma de Buenos Aires, Argentina

## Abstract

**Background:**

In October 2020, a research consortium by four HIV and infectious diseases units of general acute public hospitals of Buenos Aires city began an implementation study aimed to analyze obstacles and facilitators on telemedicine (TM) use in the care of people living with HIV(PLHIV). TM visits were conducted through phone or videocalls

**Methods:**

This research aims to analyze shifts in patient´s perception on factors that prevent and promote the utilization of telemedicine after 6 months of implementation of this strategy. We prospectively collected qualitative data through an electronic semi-structured survey delivered at and 6 months after the first teleconsultation. Surveys were anonymous and self-administered. Data was stored in Redcap® platform. These tools collected data on barriers and benefits of this strategy

**Results:**

Between October 2020 and October 2022, 45% (1871) of total enrollees (4118) answered baseline survey. Second tool was completed by 24.5% (1019) of participants. 662 participants answered both surveys. 3% (20) and 5.7% (20) in the baseline and follow-up surveys respectively, reported troubles with technological devices. This perceived barrier increased in 2.7% and this difference was statistically significant (p=0.01).

Avoiding exposures to COVID-19 and other nosocomial viruses was perceived as a benefit by 65% (1216) of patients at baseline measurement. This proportion was reduced to 57% (580) at second survey. The proportion of patients that reported avoiding commuting time as a benefit increased from 44% (823) to 52% (530) between both measurements.

**Conclusion:**

Beyond resource-limited contexts, these preliminary results illustrate a straightforward implementation of telemedicine. During COVID-19 pandemic, main benefits perceived by patients might be related to avoiding exposures to this and other viruses. After the COVID-19 sanitary situation was normalized, a higher percentage of patients reported that avoiding travel time to the hospital would be one of the main benefits of this strategy.

**Disclosures:**

**All Authors**: No reported disclosures

